# Analysis of the reduction in injury mortality disparity between urban and rural areas in developing China from 2010 to 2016

**DOI:** 10.1186/s12889-020-09027-3

**Published:** 2020-06-10

**Authors:** Yao Li, Miao Pu, Yaping Wang, Tienan Feng, Chenghua Jiang

**Affiliations:** 1 Department of Disaster and Emergency Medicine, Shanghai East Hospital, Tongji University, 1239 Siping Road, Shanghai, 200092 China; 2 Hongqiao International Institute of Medicine, Shanghai Tongren Hospital/Clinical Research Institute, Shanghai Jiao Tong University School of Medicine, 227 Chongqing South Road, Shanghai, 200025 China

**Keywords:** Injury mortality, Cause, Urban-rural disparity, Trend, China

## Abstract

**Background:**

Injuries are of growing public health concern in China, and the trends of urban-rural injury mortality disparity for the last decade are still being explored. This study aims to analyze trends in injury mortality disparity between urban and rural areas of China by region, sex, and age from 2010 to 2016.

**Methods:**

Using data from the Disease Surveillance Points system (DSPs) collected by the Chinese Center for Disease Control and Prevention (CDC) from 2010 to 2016, injury age-standardized mortality rates (ASMRs) and rate ratios (RRs) were calculated for different groups. Chi-square tests were used to compare differences in rates between urban and rural residents. The time trends of injury ASMRs were assessed via the annual percentage change (APC), and RRs were used to analyze urban-rural mortality disparity.

**Results:**

The crude injury mortality rate of rural areas was 1.5 times higher than that of urban areas. The urban-rural RR of injury ASMR decreased from 1.8 to 1.5 (APC = 5.0%) over time, from 2.0 to 1.7 (APC = 4.7%) for eastern regions, from 1.9 to 1.5 (APC = 5.4%) and from 1.6 to 1.3 (APC = 4.5%) among males and females, respectively. Further decreases were from 2.0 to 1.4 (APC = 7.8%), from 1.9 to 1.6 (APC = 6.4%), and from 1.8 to 1.2 (APC = 5.7%) in the 5–14, 45–64, and 65+ year age groups, respectively. The urban-rural RRs of ASMRs for fall, drowning and suicide decreased from 1.3 to 1.2 (APC = − 3.0%), from 2.3 to 1.6 (APC = − 13.8%) and from 2.1 to 1.6 (APC = − 9.9%,), respectively.

**Conclusions:**

The urban-rural injury mortality disparity was large, but showed a significant decreasing trend in China. Residents of eastern regions, males/females, 5–14/45+ year age groups in the urban-rural injury mortality disparity all decreased gradually during the investigated period.

## Background

Injuries have become a major public health concern worldwide. According to the World Health Organization (WHO), the global injury mortality rate was 8.6% in 2016 and the number of injury-related deaths was 4.9 million, 29% of which were caused by road-traffic accidents [1]. Low- and middle-income countries (LMICs) suffer most of the burden of these injury-related deaths, with as much as 93% of road-traffic deaths occurring in LMICs, whereas vehicles-related deaths account for only about 60% of the world’s total [2]. In addition, these countries account for 79, 90, and 80% of deaths from suicides, drowning, and falls worldwide, respectively [3–5]. The WHO pointed out that 5.1 million deaths were caused by injuries across the world in 1990 and the resulting mortality is expected to increase by 65% (to 8.4 million deaths) by 2020 because of the increase in population and changes in the age structure of society [6]. Therefore, injury prevention should be a focus of global attention, especially in LMICs.

China’s rapid economic development has caused urbanization, motorization, and changes in both the environment and lifestyle; consequently, injury has become the fifth leading cause of death in recent years [7]. The Chinese government has incorporated production safety and road traffic safety into relevant policies and regulations. In addition, national policies, such as the Outline for the Development of Chinese Children and Healthy China 2030, have clearly introduced forward requirements and related content for the prevention of injuries. The per capita income (33,616 yuan), the number of health personnel (10.8 per 1000 residents) and beds (8.4 per 1000 residents) in medical institutions in urban areas were 2.7, 2.7, and 2.2 times higher than the respective figures for rural areas in 2016 [8]. The injury mortality rate is usually higher in rural areas than in urban areas. A Chinese study indicated that the urban-rural injury mortality disparity increased from 2004 to 2010 and that the number of injury-related deaths in rural areas was almost three times higher than in urban areas [9]. However, few studies reported the trends in injury mortality disparity between urban and rural areas for the last decade that witnessed tremendous change in China. In addition, differences in injury mortality rates and causes of injuries between urban and rural areas have been reported for Ireland [10], Australia [11] and South Africa [12]. High-income countries often have national systems in place to collect information on the causes of death, while most LMICs do not have such systems [13]; therefore, there are relatively few studies on the causes of death in LMICs. The Disease Surveillance Points system (DSPs), headed by the Chinese Ministry of Health, was implemented to collect birth and death information nationwide, including injury-related deaths.

This study aims to describe the rates and the five leading causes of injury-related deaths in urban and rural areas and analyze trends in urban-rural injury mortality disparity by region, sex and age of casualties in China from 2010 to 2016.

## Methods

### Data source

Demographic and injury-related death data were obtained from the National Death Cause Surveillance Data Set between 2010 and 2016, compiled by the Chinese Center for Disease Control and Prevention (CDC) collected through the DSPs [14]. In 2013, the number of monitoring stations in the DSPs increased from 161 to 605, and evaluated 31 provinces (including autonomous regions and municipalities) in mainland China using the multi-stage stratified cluster random sampling method. With the implementation of this improvement, the percentage of the covered national population increased from 6 to 24%.

The practical definition of injury-related death that is commonly used to identify cases was based on the chapters V01 to Y98 of the International Classification of Diseases-10th Revision (ICD-10), specific coding by injury is provided in Additional file 1. These causes of injury were the underlying causes of death. This study reports the five major causes that account for more than 80% of all injury-related deaths, including traffic injury, fall, suicide, drowning and poisoning. Data is divided into unintentional injuries (traffic injury, poisoning, fall, fire, drowning, and others) and intentional injuries (suicide, homicide, and others). The DSPs defines all counties (including county-level cities) as rural areas and all districts of a city as urban areas, based on the relevant administrative division. Thirty-one provincial administrative regions (provinces, municipalities, and autonomous regions) in mainland China were divided into eastern, central and western regions according to their geographical location (see Additional file 2). The data from urban and rural areas were stratified by age (0, 1–4, 5–14, 15–44, 45–64, and 65+ years of age). In practice, this study used unadjusted data, although the true mortality rates are likely to have been under-estimated in the DSPs.

### Monitoring content

The various types of medical institutions linked with the monitoring stations are responsible for submitting cause-of-death information reports. The medical certificate of death can only be issued by medical personnel with professional qualifications as physicians. Next, deaths in monitoring stations are reported by trained doctors through an online death registration and reporting system. Finally, CDC death report management personnel reviews the death information reported within their jurisdiction every working day. The death certificate mainly includes basic information of the deceased person, information concerning the death and ICD-10 coding [14].

### Data analysis

Epidata 3.1 was used for data entry and Microsoft Excel 2016 and SPSS 20.0 statistical software were used for data management and analysis. Injury crude mortality rates and age-standardized mortality rates (ASMRs) (to investigate chronological trends) per 100,000 population (and 95% confidence intervals (CIs)) were calculated for Chinese urban and rural residents between 2010 and 2016. The standard used to calculate adjusted rates was the sum of the populations obtained from the 2010 Chinese Census by age. Rate ratios (RRs) and 95% CIs corresponding to urban and rural categories were calculated by using the urban category as reference, thus reflecting the disparity between urban and rural residents.

Basic information on injury deaths, compared rates and causes of injury deaths in urban and rural areas were described by region (eastern, central and western), sex, and age, and injury ASMRs and RR (i.e., rural-urban ratios) were calculated for different groups to analyze mortality time trends and urban-rural mortality disparity. The Chi-square tests were used to analyze differences in injury mortality rates between urban and rural areas for the various investigated groups. When the assumptions for the Chi-square test was violated, the Chi-square test was replaced by the Fisher’s exact test. In addition, the time trend of injury ASMRs were assessed from the APC by t-test. *p* < 0.05 was considered statistically significant.

Formula and calculations: $\mathrm{ASMR}=\frac{\sum {nP}_x\times {nM}_x}{\sum {nP}_x}$, where nPx represents the age-specific population of the standard population, nMx represents the age-specific mortality rate of the population to be standardized, n represents the interval of each age group, and x represents the starting age of each age group.

$\mathrm{RR}=\frac{rate\  per\ {injury\ type}_{rural}}{rate\  per\ {injury\ type}_{urban}}$. y = *α + βx*, where y represents ln(ASMR), x represents the year. To fit the linear regression model, *β* was used as the regression coefficient. APC = 100% × (exp(*β*)-1).

## Results

### Basic information on injury deaths for China, 2010–2016

According to the DSPs, injury deaths (n = 615,498) accounted for 7.8% of all deaths (n = 7,882,538). Urban injury deaths (n = 153,348) accounted for 6.2% of all deaths in urban areas (n = 2,471,056), whereas rural injury deaths (n = 462,150) accounted for 8.5% of all deaths in rural areas (n = 5,411,482) over the investigated period. The urban-rural ratio of population number and injury-related deaths was 2.0 and 3.0 between 2010 and 2016, respectively (Table 1).

**Table 1:** Total people surveyed, deaths and injury deaths in urban and rural areas of China, 2010–2016

	2010		2011		2012		2013		2014		2015		2016	
	Number(%)	Urban-Rural ratio	Number(%)	Urban-Rural ratio	Number(%)	Urban-Rural ratio	Number(%)	Urban-Rural ratio	Number(%)	Urban-Rural ratio	Number(%)	Urban-Rural ratio	Number(%)	Urban-Rural ratio
Deaths caused by injury	40,321	2.7	39,448	2.9	38,731	2.9	117,088	3.3	126,035	3.1	124,621	3	129,254	2.9
Urban	10,922 (27.1%)		10,129 (25.7%)		9951 (25.7%)		27,422 (23.4%)		30,579 (24.3%)		31,210 (25.0%)		33,135 (25.6%)	
Rural	29,399 (72.9%)		29,319 (74.3%)		28,780 (74.3%)		89,666 (76.6%)		95,456 (75.7%)		93,411 (75.0%)		96,119 (74.4%)	
Total number of deaths	453,211	1.8	445,477	1.9	459,836	1.9	1,463,851	2.4	1,643,377	2.3	1,673,245	2.2	1,743,541	2.2
Urban	161,163 (35.6%)		154,677 (34.7%)		160,573 (34.9%)		435,497 (29.8%)		499,019 (30.4%)		515,465 (30.8%)		544,662 (31.2%)	
Rural	292,048 (64.4%)		290,800 (65.3%)		29,926,365.1%)		1,028,354 (70.2%)		1,144,358 (69.6%)		1,157,780 (69.2%)		1,198,879 (68.8%)	
Total people surveyed	78,766,626	1.6	77,396,478	1.5	77,215,997	1.5	227,236,284	2.2	253,610,895	2.1	257,571,377	2.1	264,772,868	2
Urban	29,813,874 (37.9%)		30,848,186 (39.9%)		30,767,220 (39.8%)		70,300,058 (30.9%)		80,963,448 (31.9%)		82,930,744 (32.2%)		88,469,225 (33.4%)	
Rural	48,952,752 (62.1%)		46,548,292 (60.1%)		46,448,777 (60.2%)		156,936,226 (69.1%)		172,647,447 (68.1%)		174,640,633 (67.8%)		176,303,643 (66.6%)	

### Epidemiological characteristics of injury-related deaths in urban and rural areas

**Fig. 1:**
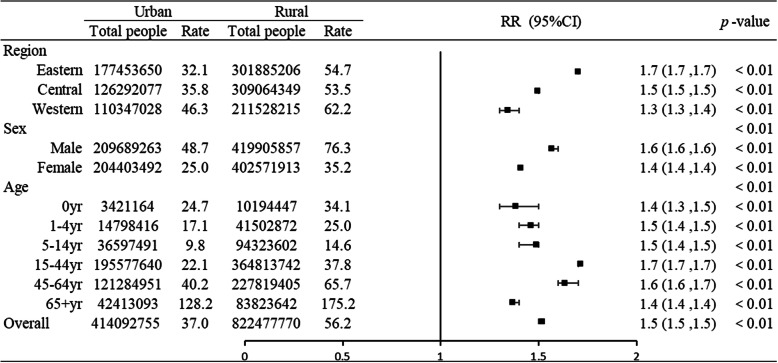
Ratio rate for injury mortality rates among urban and rural residents by region, sex and age in China, 2010–2016. Ratio rate (RR) was calculated using the injury mortality rate of the urban population as the referent, RR and 95% confidence intervals (CI) reflected the disparity between urban and rural residents

**Fig. 2:**
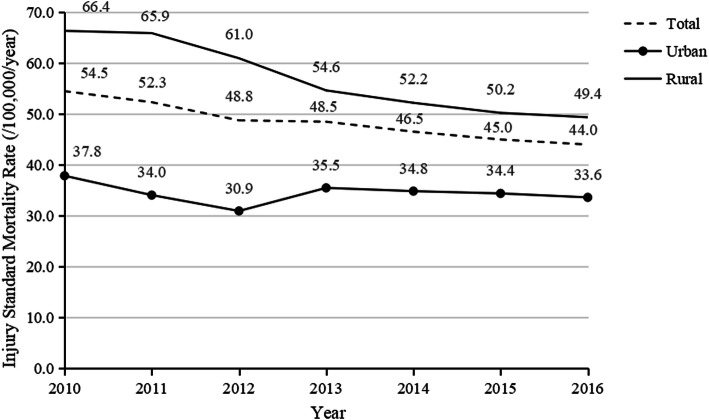
Age-standardized injury mortality rates per 100,000 among urban and rural residents in China, 2010–2016

The crude injury mortality rate for the total surveyed population was 49.8 (95% CI: 49.7, 49.9) per 100,000. That of rural residents [56.2 per 100,000 (95% CI: 56.0, 56.4)] was significantly higher than that of urban residents [37.0 per 100,000 (95% CI: 36.9, 37.2)] (*p* < 0.01); RR = 1.5 (95% CI: 1.5, 1.5) between 2010 and 2016. The crude injury mortality rates in rural areas were all higher than that of urban areas, stratified by region, age, and sex during the study period (*p* < 0.01) (Fig. 1). The injury ASMR among the total population and rural residents from 2010 to 2016 decreased by 19.3% (APC = − 3.5%, *p* < 0.01) and 25.6% (APC = − 5.5%, *p* < 0.01); however, the difference among urban residents was not statistically significant (APC = − 0.8%, *p* = 0.6) (Fig. 2). The injury mortality disparity between urban and rural areas decreased over time, and the RR of injury ASMR decreased from 1.8 to 1.5 (APC = 5.0%, *p* < 0.05).

**Fig. 3:**
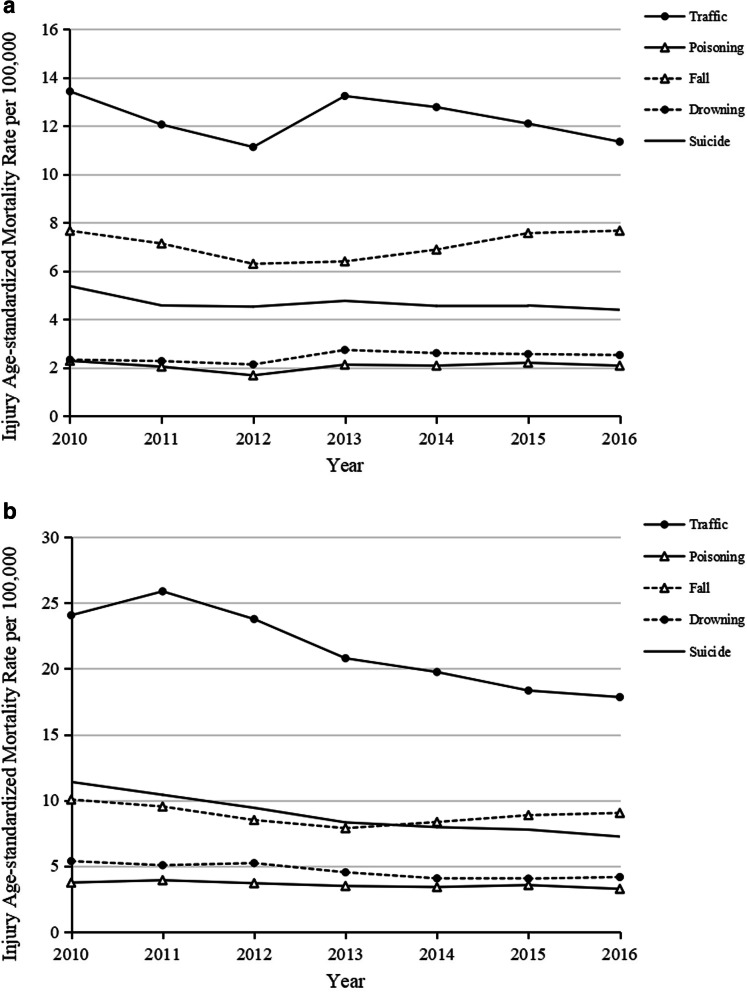
Age-standardized mortality rates per 100,000 for the five leading causes of injury-related deaths among urban (a) and rural (b) residents in China, 2010–2016

The five leading causes of injury-related deaths in urban and rural areas were traffic injury, fall, suicide, drowning, and poisoning, which accounted for more than 80% of all injury-related deaths. The mortality rates for the five causes in rural areas were all higher than in urban areas (*p* < 0.01) (Table 2). Comparing the urban-rural disparity of the mortality rates for the five causes, drowning [RR = 2.1 (95% CI: 2.0, 2.1)] was the largest cause of death and fall was the smallest [RR = 1.2 (95% CI:1.2, 1.2)]. In rural areas, the ASMRs of traffic injury, poisoning, drowning, and suicide gradually decreased by 25.9% (APC = − 6.1%, *p* < 0.01), 12.9%(APC = − 2.5%, *p* < 0.05), 22.6%(APC = − 5.2%, *p* < 0.01) and 36.5% (APC = − 7.2%, *p* < 0.05), respectively. In urban areas, differences in the five causes over time were not statistically significant (Fig. 3). The urban-rural disparity in ASMRs of fall, drowning, and suicide became smaller over time, and RRs of cause-specific ASMRs decreased from 1.3 to 1.2 (APC = − 3.0%, *p* < 0.01), 2.3 to 1.6 (APC = − 13.8%, *p* < 0.05) and 2.1 to 1.6 (APC = − 9.9%, *p* < 0.01).

**Table 2:** The five leading causes of injury mortality rates (95%CI) per 100, 000 among urban and rural residents by region, sex and age in China, 2010–2016

	Urban					Rural				
	Traffic injury	Poisoning	Fall	Drowning	Suicide	Traffic injury	Poisoning	Fall	Drowning	Suicide
Region										
Eastern	10.8 (10.6, 10.9)	1.7 (1.7, 1.8)	7.5 (7.3, 7.6)	1.8 (1.8, 1.9)	4.6 (4.5, 4.7)	20.6 (20.5, 20.8)	2.8 (2.7, 2.8)	10.6 (10.5, 10.7)	3.8 (3.7, 3.9)	7.6 (7.5, 7.7)
Central	13.1 (12.9, 13.3)	2.2 (2.1, 2.3)	6.4 (6.3, 6.6)	2.5 (2.5, 2.6)	5.8 (5.7, 6.0)	19.9 (19.7, 20.0)	3.3 (3.3, 3.4)	7.4 (7.3, 7.4)	4.4 (4.3, 4.5)	10.4 (10.2, 10.5)
Western	16.6 (16.3, 16.8)	3.2 (3.1, 3.3)	10.6 (10.4, 10.8)	3.5 (3.4, 3.6)	4.8 (4.7, 5.0)	21.4 (21.2, 21.6)	5.2 (5.1, 5.3)	11.1 (11.0, 11.2)	5.6 (5.5, 5.7)	7.9 (7.8, 8.0)
Sex										
Male	18.9 (18.7, 19.1)	3.1 (3.1, 3.2)	9.3 (9.2, 9.5)	3.2 (3.1, 3.3)	5.7 (5.6, 5.8)	30.6 (30.4, 30.8)	5.0 (5.0, 5.1)	11.7 (11.6, 11.8)	5.9 (5.8, 6.0)	9.8 (9.7, 9.9)
Female	7.1 (6.9, 7.2)	1.3 (1.3, 1.4)	6.6 (6.4, 6.7)	1.7 (1.7, 1.8)	4.3 (4.2, 4.4)	10.1 (10.0, 10.1)	2.1 (2.1, 2.2)	7.2 (7.1, 7.3)	3.0 (3.0, 3.1)	7.6 (7.5, 7.7)
Age										
0 yr	3.2 (2.6, 3.8)	0.4 (0.2, 0.7)	1.7 (1.3, 2.1)	1.3 (0.9, 1.7)	0.0 (0.0, 0.0)	3.7 (3.3, 4.1)	1.0 (0.8, 1.2)	1.8 (1.6, 2.1)	2.0 (1.8, 2.3)	0.0 (0.0, 0.0)
1-4 yr	4.8 (4.5, 5.2)	0.7 (0.5, 0.8)	2.0 (1.8, 2.2)	6.6 (6.2, 7.0)	0.0 (0.0, 0.0)	6.6 (6.3, 6.8)	1.0 (0.9, 1.1)	1.9 (1.8, 2.1)	11.1 (10.8, 11.5)	0.0 (0.0, 0.0)
5-14 yr	3.1 (2.9, 3.3)	0.4 (0.3, 0.4)	0.9 (0.8, 1.0)	3.8 (3.6, 4.0)	0.4 (0.4, 0.5)	4.1 (3.9, 4.2)	0.6 (0.6, 0.7)	1.0 (0.9, 1.0)	6.6 (6.4, 6.8)	0.5 (0.4, 0.5)
15-44 yr	10.1 (10.0, 10.2)	1.6 (1.5, 1.7)	2.2 (2.1, 2.3)	1.5 (1.4, 1.5)	2.9 (2.8, 3.0)	17.9 (17.7, 18.0)	2.6 (2.6, 2.7)	3.3 (3.2, 3.3)	2.6 (2.6, 2.7)	4.7 (4.6, 4.7)
45-64 yr	17.1 (16.8, 17.3)	2.9 (2.8, 3.0)	5.9 (5.8, 6.1)	1.9 (1.8, 2.0)	6.1 (6.0, 6.3)	28.0 (27.8, 28.2)	4.7 (4.6, 4.8)	8.8 (8.6, 8.9)	3.2 (3.1, 3.3)	10.7 (10.6, 10.9)
65 + yr	27.3 (26.9, 27.8)	5.7 (5.5, 5.9)	49.0 (48.4, 49.7)	6.2 (6.0, 6.4)	17.9 (17.5, 18.3)	39.3 (38.8, 39.7)	9.9 (9.7, 10.2)	52.9 (52.4, 53.4)	10.9 (10.7, 11.2)	35.6 (35.2, 36.0)
Overall	13.0 (12.9, 13.2)	2.3 (2.2, 2.3)	8.0 (7.9, 8.1)	2.5 (2.4, 2.5)	5.0 (5.0, 5.1)	20.5 (20.4, 20.6)	3.6 (3.6, 3.7)	9.5 (9.4, 9.6)	5.2 (5.1, 5.2)	8.7 (8.6, 8.8)

### Regional distribution of injury-related deaths in urban and rural areas

**Fig. 4:**
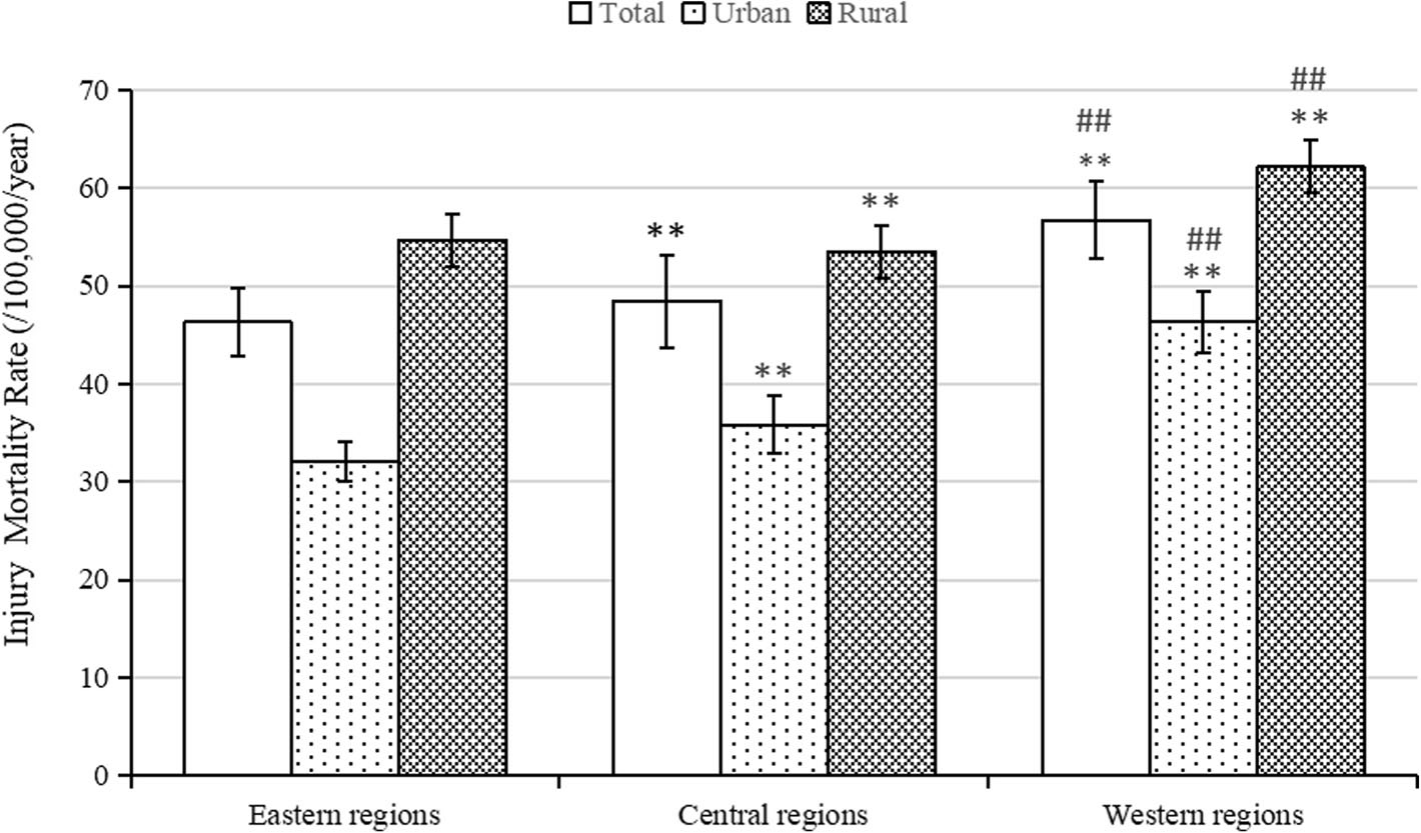
Injury mortality rates per 100,000 by region among urban and rural residents in China, 2010–2016. Distribution of rates significantly different by Chi-square test. Compared to eastern regions, **p<0.01; Compared to central regions, ##*p*<0.01

The injury mortality rate increased gradually from east to west among urban residents (*p* < 0.01) (Fig. 4). The disparity in injury mortality between urban and rural residents decreased gradually from east to west (Fig. 1). For rural residents, the injury ASMR in eastern, central, and western regions decreased by 30.0% (APC = − 6.1%), 26.1% (APC = − 6.0%), and 16.7% (APC = − 3.4%), respectively (all *p* < 0.01). However, the differences in urban residents by region were not statistically significant over time. The disparity between urban and rural areas became smaller in eastern regions, and the RR of injury ASMR decreased from 2.0 to 1.7 (APC = 4.7%, *p* < 0.01), although urban-rural differences were small in the other two regions from 2010 to 2016.

The five leading causes of injury mortality in rural residents by region were higher than for urban residents (*p* < 0.01) (Table 2). Drowning was the cause of death with the largest urban-rural injury mortality disparity [RR = 2.1 (95% CI: 2.0, 2.2)] among eastern residents, whereas suicide was the cause of death among central and western residents [RR = 1.8 (95% CI: 1.7, 1.8), 1.6 (95% CI: 1.6, 1.7)]. Fall was the cause of the smallest urban-rural disparity in injury deaths, with RRs of 1.4 (95% CI: 1.4, 1.4), 1.1 (95% CI: 1.1, 1.2), and 1.1 (95% CI: 1.0, 1.1) for eastern, central, and western regions, respectively. For rural residents, the ASMRs of traffic injury, suicide, and drowning showed a decreasing trend in eastern (APC = − 6.0, − 6.8%, and − 5.9%, respectively), central (APC = − 6.8, − 9.7%, and 6.1%, respectively), and western regions (APC = − 5.4, − 3.0%, and − 6.4%, respectively). For urban residents, the ASMR of traffic injury in eastern regions and of suicide in western regions gradually decreased by 27.00% (APC = − 3.9%, *p* < 0.05) and 30.5% (APC = − 5.8%, *p* < 0.05), respectively. In contrast, the ASMR of fall in western regions increased by 19.3% (APC = 3.2%, *p* < 0.05) from 2010 to 2016.

### Distribution of injury-related deaths by sex in urban and rural areas

The injury mortality rate of urban and rural residents by sex is shown in Fig. 1. Urban-rural differences in male injury mortality rates exceeded those of females (Fig. 1). The injury ASMR among males and females in rural areas gradually decreased by 25.8% (APC = − 5.7%, *p* < 0.01) and by 25.8% (APC = − 5.2%, *p* < 0.01), respectively. However, differences among urban residents by sex were not statistically significant. The urban-rural disparity in sexes both decreased over time, and RRs of injury ASMR among males and females decreased from 1.9 to 1.5 (APC = 5.4%, *p* < 0.05) and from 1.6 to 1.3 (APC = 4.5%, *p* < 0.05), respectively.

The injury mortality rates for the five leading causes by sex were higher among rural residents than among urban residents (*p* < 0.01) (Table 2). Drowning was the main cause of urban-rural injury mortality disparity [RR = 1.9 (95% CI: 1.8,1.9)] among male residents, whereas it was suicide among female residents [RR = 1.8 (95% CI: 1.7,1.8)]. Fall was the cause of the smallest urban-rural disparity in males [RR = 1.3 (95% CI:1.2, 1.3)] and females [RR = 1.1 (95% CI:1.1, 1.1)]. In rural areas, the ASMRs of traffic injury, suicide, and drowning showed a decreasing trend among males (APC = 6.6, − 6.8%, and − 5.2%) and females (APC = − 4.3, − 8.3%, and − 5.1%) (all *p* < 0.05), respectively. In urban areas, the ASMR of suicide among female residents gradually decreased by 19.7% (APC = − 2.8%, *p* < 0.05).

### Distribution of injury-related deaths by age in urban and rural areas

The lowest injury mortality rates in urban and rural areas were found in the 5–14-year age group, while the highest rates were found in the 65+ year age group. The smallest gap in injury mortality between urban and rural residents was identified in the 65+ year age group and the largest gap in the 15–44-year age group (Fig. 1). Injury mortality rates between 2010 and 2016 showed a gradual decreasing trend in the 0, 1–4, 5–14, and 15–44-year age groups in rural areas, whereas it increased in the 65+ year age group in urban areas (Table 3). The urban-rural disparity became smaller in the 5–14, 45–64, and 65+ year age groups from 2010 to 2016, and RRs of injury mortality decreased from 2.0 to 1.4 (APC = 7.8%, *p* < 0.05), from 1.9 to 1.6 (APC = 6.4%, *p* < 0.05), and from 1.8 to 1.2 (APC = 5.7%, *p* < 0.05), respectively.

**Table 3:** Age-standardized mortality rates per 100,000 for injuries by age among urban and rural residents of China, 2010–2016

Age	Year							APC (95%CI)	*p*-value
	2010	2011	2012	2013	2014	2015	2016		
Urban									
0 yr	25.8	28.1	19.4	27.4	22.5	25.8	23.7	−1.0% (−7.8, 5.8%)	0.7
1-4 yr	16.9	18.4	14.7	19.7	18.1	16.5	15.1	−1.2% (−6.8, 4.4%)	0.6
5-14 yr	9.2	9.8	9.8	10.9	10.3	9.7	8.9	−0.3% (−3.8, 3.2%)	0.9
15-44 yr	27.4	22.3	19.7	25.6	23.2	20.7	18.7	−3.9% (−9.7, 1.9%)	0.1
45-64 yr	39.3	34.3	37.7	41.3	40.8	40.9	41.5	2.1% (−0.6, 4.8%)	0.1
65 + yr	113.6	112.4	111.6	122.3	128.2	137.7	137.9	4.1% (2.4, 5.8%)	**< 0.01**
Rural									
0 yr	39.6	39.6	41.4	39.4	34.8	29.0	28.9	−6.0% (−10.1, −2.0%)	**0.0**
1-4 yr	30.1	35.3	33.6	26.1	24.6	21.5	21.2	−8.1% (−13.1, − 3.0%)	**< 0.01**
5-14 yr	18.2	19.3	19.2	15.4	13.9	13.0	12.6	−7.6% (−11.3, −3.9%)	**< 0.01**
15-44 yr	45.0	47.4	45.9	40.4	38.0	33.9	31.7	−6.6% (−9.3, −3.9%)	**< 0.01**
45-64 yr	74.4	82.1	73.5	66.9	63.0	61.0	64.7	−4.1% (−7.1, −1.1%)	**0.0**
65 + yr	202.2	169.3	180.6	171.3	170.8	180.5	172.2	−1.5% (−4.3, 1.3%)	0.3

There were no significant differences in cause-specific mortality rates between specific age groups in urban and rural areas, including traffic injury as well as fall in the 0-year age group, fall in the 1–4-year age group, and fall as well as suicide in the 5–14-year age group. However, other injury-related causes of mortality in rural areas were higher than in urban areas by age (*p* < 0.05) (Table 2). Poisoning was the cause of the largest urban-rural injury mortality disparity and drowning was the smallest disparity in the 0-year age group [RR = 2.4 (95% CI:1.4, 4.1), 1.5 (95% CI: 1.1, 2.1)]. Drowning was the largest disparity and traffic injury was the smallest disparity among the 1–4-year [RR = 1.7 (95% CI: 1.6, 1.8), 1.3 (95% CI: 1.2, 1.4)] and 5–14-year age groups [RR = 1.7 (95% CI: 1.6, 1.8), 1.3 (95% CI: 1.2, 1.4)]. Traffic injury was the largest disparity in the 15–44-year age group [RR = 1.8 (95% CI: 1.7, 1.8)] and suicide was the largest disparity in the 45–64-year and 65+ year age groups [RR = 1.8 (95% CI: 1.7, 1.8), 2.0 (95% CI: 1.9, 2.0)]. In contrast, fall was all the cause with the smallest disparity in these three age groups [RR = 1.5 (95% CI: 1.4, 1.5), 1.5 (95% CI: 1.4, 1.5), and 1.1 (95% CI: 1.1, 1.1)]. In rural areas, the ASMRs of traffic injury and suicide gradually decreased in the 5–14-year (APC = − 6.3% and − 10.9%), 15–45-year (APC = − 7.9% and − 5.8%), 45–64-year (APC = − 5.4% and − 4.8%) and 65+ year age groups (APC = − 2.0% and − 7.9%) and the ASMR of drowning in the 5–14 (APC = − 11.0%) and 15–45-year groups (APC = − 10.0%) also gradually decreased. In urban areas, it is noteworthy that the mortality rates of fall and drowning increased by 26.2 and 31.74% in the 45–64-year age group (APC = 5.7 and 5.8%), as well as by 41.7 and 37.3% in the 65+ year age group (APC = 6.9 and 6.8%), respectively (all *p* < 0.05).

## Discussion

This study investigated trends in urban-rural injury mortality disparity for China from 2010 to 2016 and found that this disparity decreased substantially. The urban-rural RR of injury ASMR decreased from 1.8 to 1.5, in which the rate of rural residents decreased by 25.6% and that of urban residents did not significantly change.

These findings show that the decline in rural injury mortality lead to a narrowing of urban-rural disparity, which was inconsistent with the results reported by Zhang et al. (2014) [9] and Ding et al. (2018) [15]. Zhang et al. (2014) reported that a widening urban-rural disparity resulted from a reduction of 25.9% in urban injury mortality in China from 2004 to 2010, while Ding et al. (2018) reported that the urban-rural injury mortality disparity did not significantly change in Chongqing from 2011 to 2016, because mortality of urban and rural residents both remained unchanged. Studies for Ireland [10], Australia [11] and South Africa [12] identified the differences in injury mortality and causes of injury deaths between rural and urban residents, but did not account for trends in urban-rural disparity. The present study found that the mortality rates for the five leading causes were significantly higher in rural areas and the main causes of injury-related deaths were the same between rural and urban residents. However, only transport-related injury mortality rates were significantly higher in rural provinces of South Africa [12], and there were lower mortality rates in rural residents for falls and poisoning in Ireland [10]. Injury mechanisms differed between rural and urban population in Australia [11] and South Africa [12].

The large gap in injury mortalities between urban and rural residents of China could be due to insufficient emergency resources in rural areas of China, which results in longer arrival times and difficulties in transporting injured patients [16]. The number of emergency institutions and personnel was 1.5 and 1.8 times higher in urban areas than in rural areas, respectively, during the period of 2011–2015. The proportion of urban residents arriving at the nearest medical institution within 15 min was 87.8%, whereas only 80.2% of rural residents arrived within 15 min [17, 18]. Another reason may be the difference in urban and rural protective infrastructure [7, 16].

Fortunately, the narrowing of urban-rural disparity was the result of the decreased rural injury mortality, which may be closely related to the overall environment of rural rapid development of the economy, education and medical care under the construction of the new socialist countryside [7]. Clearly, the number of emergency institutions and personnel has witnessed a steady growth trend and the growth rate in rural areas has been faster than in urban areas [17, 18]. Moreover, improved infrastructure projects in rural areas, including bridges, roads, and dikes, may also decrease exposure to common causes of injury [7, 16]. The urban-rural disparity for drowning, fall, and suicide has decreasing every year. Firstly, experts believe that population with high drowning risk in rural China is caused by left-behind children aged 0–14 who swim unattended in lakes or rivers [19, 20]. This differs from the characteristics of drowning incidents in Japan (which mainly affects older adults involving bathtubs) and in the USA (which mainly affects children aged 0–14 years in swimming pools) [21]. The number of rural left-behind children in 2016 (17.3 million) decreased by 23.8% compared with 2012 (22.7 million), which may have contributed to the decrease in drowning mortality in rural areas [19, 20]. Secondly, most fatal falls occurred in the elderly aged 65 years worldwide [5], the fall mortality rate among urban residents increased by 41.7% in the 65+ year-old age group, while rural residents retained stable rates over time. The higher death rates of fall in urban areas may be due to population aging, as well as the environment and physical conditions in the elderly [16]. Finally, restricting access to drugs or compounds used to commit suicide and providing medication for mental disorders (especially depression) can decrease the risk of suicide. This has proved to be an effective intervention in LMICs [22, 23], and studies for China and India have identified easy access to highly toxic pesticides as an important risk factor for suicide in rural areas [24–27]. In recent years, China has restricted regional sales and even banned several types of highly toxic pesticides, a measure that may have indirectly reduced the rates of suicide in rural areas [28].

The highest injury mortality rate among urban and rural residents was both in the western regions, the rate for urban and rural residents of Guangxi [29] and Chongqing [15] (western regions) was higher than for those in Beijing [30] and Shanghai [31] (eastern regions), and in Hubei [16] (central region). This may be because the number of emergency facilities in eastern regions is almost twice the sum total of that of the other two regions and the urban and rural areas of western regions have the lowest number, which affects the coverage and response time of injury emergency services [17]. The gap in injury mortality rates between urban and rural residents was largest in eastern regions and smallest in western regions, which may be due to the disparity of the economic development, which indirectly leads to differences in medical emergency and protective infrastructures between regions [9]. Economic development and urbanization rates are highest in eastern regions, but this is also where the gap between urban and rural areas is largest [17, 32]. Given the uneven distribution of existing medical resources and the poor knowledge of first-aid measures by the general Chinese population, first-aid training should be provided to residents through community or village committees or relevant medical information technology should be introduced to decrease injury-related mortality [33]. It should be noted that the fall mortality rate increased for western urban residents between 2010 and 2016, indicating that fatal falls have not decreased in high-risk elderly groups. Therefore, residents of western urban areas should be made aware of fall prevention strategies. In addition, eastern rural areas should strengthen drowning prevention measures, while central and western rural areas should focus on suicide prevention. Drowning accounted for the largest disparity in injury deaths between urban and rural residents of the eastern regions and suicide did so for both the central and western regions.

The injury mortality rate for males in urban and rural areas was higher than for females, which was mainly because the mortality rate associated with traffic injuries and drowning for males living in urban and rural areas was much higher than for females. Relevant studies conducted in China [34], India [35], Tanzania [36], and Rwanda [37] showed that rural residents and males were high-risk groups for traffic-related injury deaths because of poor road conditions and more dangerous driving behavior by males. According to the WHO, the drowning mortality rate in males is twice that of females because more males work in aquatic environments such as lakes or the sea [38]. In the case of China, males may be more involved in activities in uncontrolled rural aquatic environments and participate more frequently in outdoor activities [39]. It should be noted that the suicide mortality rate in females was close to that of males in rural areas. The suicide death rate for males is often much higher than for females in rural areas of most countries, since males in rural areas are more likely to choose violent suicides by means of firearms, which are difficult to obtain in China because they are illegal. Evidence suggests that a low educational level, low income, and mental disorders are associated with suicide among rural females and the most common method of committing suicide is by ingestion of highly toxic pesticides in China [28, 40]. The reason for the small sex difference in suicide mortality in rural China may be that highly toxic pesticides are readily available to both males and females [41]. To conclude, prevention strategies should focus on drowning in urban males and suicide in rural females.

With the serious aging problem currently affecting China, the proportion of the elderly who live alone in rural areas is continuously increasing. Lower social security, declining health, and poor spiritual life can increase the risk of suicide in rural middle and advanced-aged persons, explaining the wide gap in suicide death rates between urban and rural residents over 45 years of age [42]. Different prevention measures should be implemented for residents of different age in urban and rural areas. Prevention of drowning and suicide in adolescents can be accomplished by publicity in schools and by specifically directed mental health education. The elderly should be monitored by family members in case of fall and they should receive help to adapt to age-related physical, sensory, and cognitive changes. Finally, village committees should provide more extensive psychological counseling and health education for rural women and the elderly and conduct psychological interventions in high-risk groups if necessary.

This study has several limitations: First, this study did not adjust for potentially under-estimated injury mortality rates in the DSPs. Second, the data from DSPs did not include further details on injury deaths, which prevented the direct investigation of the potential influencing factors that contribute to the disparity between urban and rural injury mortality. Third, information bias may result from misclassifications of the causes of death.

## Conclusions

The urban-rural injury mortality disparity was large, but showed a significant decreasing trend in China. Residents of eastern regions, males/females, 5–14/45+ year age groups in the urban-rural injury mortality disparity all decreased gradually during the investigated period. Further research is needed to identify factors contributing to injury mortality in different group, and to assess prevention strategies to reduce injury and narrow the urban-rural gap in injury mortality.

## Supplementary information

**Additional file 1.** The ICD-10 codes for types of injury.

**Additional file 2.** Map of China with geographical divisions. Red: eastern region; blue: central region; white: western region. Source: Zhang L, Li Z, Li X, et al. Study on the trend and disease burden of injury deaths in Chinese population, 2004–2010. PLoS One. 2014;9(1):e85319. doi:https://doi.org/10.1371/journal.pone.0085319. This original figure is under a Creative Commons Attribution License.

## Data Availability

The data that support the findings of this study are available from the National Death Cause Surveillance Data Set between 2010 and 2016, compiled by the Chinese Center for Disease Control and Prevention (CDC) collected through the Disease Surveillance Points system.

## Abbreviations

LMICs: Low- and middle-income countries
DSPs: Disease Surveillance Points system
CDC: Center for Disease Control and Prevention
ICD-10: International Classification of Disease-10th Revision
ASMRs: Age-standardized mortality rates
RR: Rate ratios
APC: Annual percentage change
CI: Confidence interval
