# Supplementary material for: Analysis of the reduction in injury mortality disparity between urban and rural areas in developing China from 2010 to 2016
Source: BMC Public Health. 2020 Jun 10;20:903. doi: 10.1186/s12889-020-09027-3 (PMC7288693; doi:10.1186/s12889-020-09027-3)
Supplement: Supplementary file 1 — Additional file 1. The ICD-10 codes for types of injury. [file 12889_2020_9027_MOESM1_ESM.docx]

**Appendix 1** The ICD-10 codes for types of injury

| **Injury causes** | **ICD-10 codes** |
| --- | --- |
| traffic injury | V01-V99 |
| Fall | W00-W19 |
| Drowning | W65-W74 |
| Fire/burn | W32-W40 |
| animal contact or Animal and plant poisoning | W53-W59, X20-X29 |
| suffocation | W75-W84 |
| Poisoning | X40-X49 |
| Suicide | X60-X84 |
| homicide | X85-Y09 |
| War | Y35-Y36 |
| Undetermined intent | Y10-Y34 |
