# Supplementary material for: Analysis of the reduction in injury mortality disparity between urban and rural areas in developing China from 2010 to 2016
Source: BMC Public Health. 2020 Jun 10;20:903. doi: 10.1186/s12889-020-09027-3 (PMC7288693; doi:10.1186/s12889-020-09027-3)
Supplement: Supplementary file 2 — Additional file 2. Map of China with geographical divisions. Red: eastern region; blue: central region; white: western region. Source: Zhang L, Li Z, Li X, et al. Study on the trend and disease burden of injury deaths in Chinese population, 2004–2010. PLoS One. 2014;9(1):e85319. doi:https://doi.org/10.1371/journal.pone.0085319. This original figure is under a Creative Commons Attribution License. [file 12889_2020_9027_MOESM2_ESM.docx]

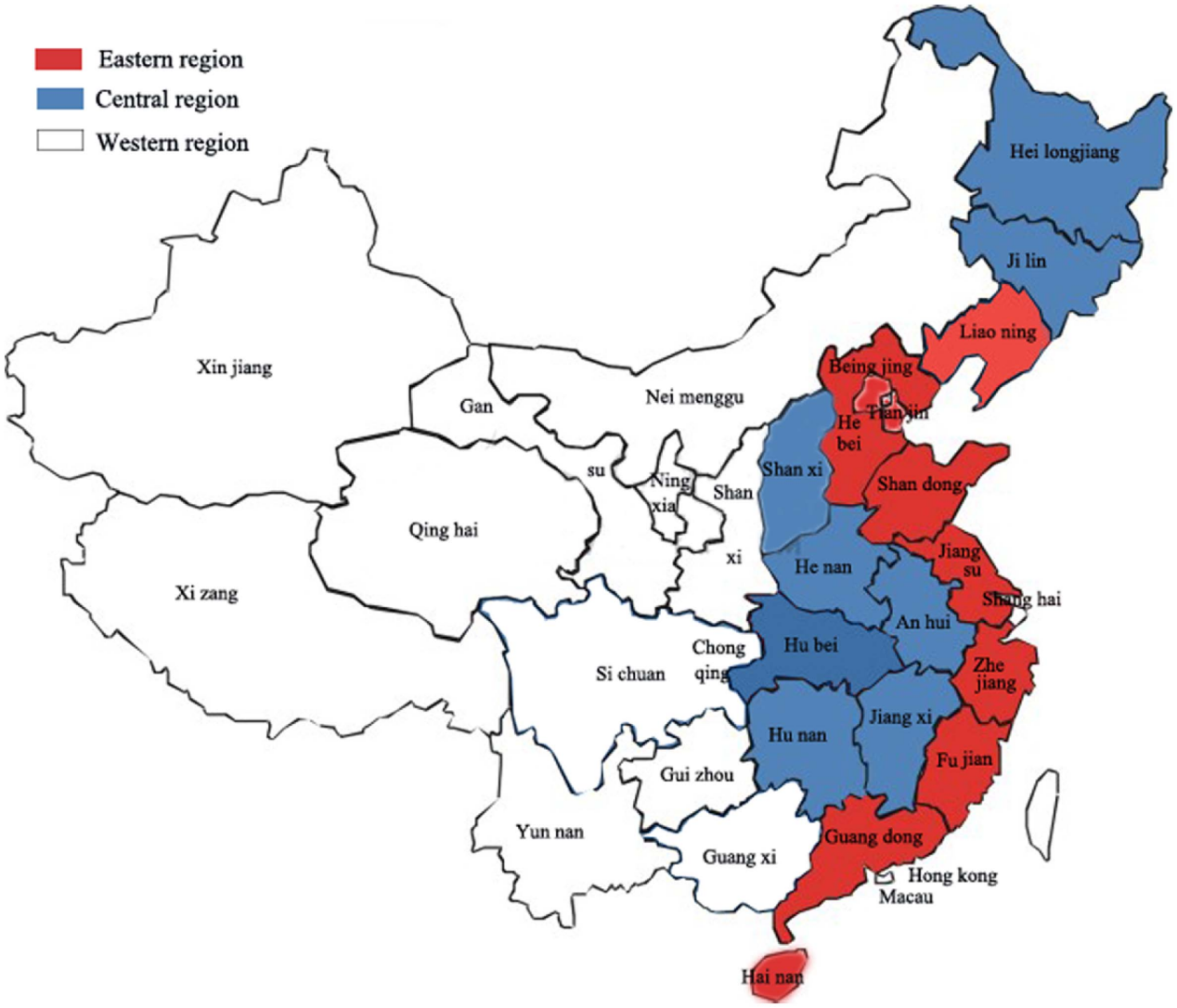


**Appendix 2** Map of China with geographical divisions. Red: eastern region; blue: central region; white: western region. Source: Zhang L, Li Z, Li X, et al. Study on the trend and disease burden of injury deaths in Chinese population, 2004-2010. PLoS One. 2014;9(1):e85319. doi:10.1371/journal.pone.0085319. This original figure is under a Creative Commons Attribution License.
